# p53 amyloid aggregation in cancer: function, mechanism, and therapy

**DOI:** 10.1186/s40164-022-00317-7

**Published:** 2022-09-28

**Authors:** Jingzhi Li, Ming Guo, Lin Chen, Zhuchu Chen, Ying Fu, Yongheng Chen

**Affiliations:** 1 Department of Oncology, NHC Key Laboratory of Cancer Proteomics & State Local Joint Engineering Laboratory for Anticancer Drugs, National Clinical Research Center for Geriatric Disorders, Xiangya Hospital, Central South University, Changsha, 410008 Hunan China; 2 Department of Obstetrics, Xiangya Hospital, Central South University, Changsha, 410008 Hunan China; 3 Molecular and Computational Biology Program, Department of Biological Sciences and Department of Chemistry, University of Southern California, Los Angeles, CA 90089 USA

**Keywords:** p53, Aggregation, Amyloid, Cancer, Mechanism

## Abstract

Similar to neurodegenerative diseases, the concept that tumors are prion like diseases has been proposed in recent years. p53, the most well-known tumor suppressor, has been extensively studied for its expression, mutation, and function in various tumors. Currently, an interesting phenomenon of p53 prion-like aggregation has been found in several tumors, and studies have found that its pathological aggregation may lead to functional alterations and ultimately affect tumor progression. It has been demonstrated that the mechanism of p53 aggregation involves its mutation, domains, isoform, etc. In addition to p53 itself, some other factors, including Zn^2+^ concentration, pH, temperature and chaperone abnormalities, can also contribute to p53 aggregation. Although there are some studies about the mechanism and role of p53 aggregation and amyloidosis in tumors, there still exist some controversies. In this paper, we review the mechanism of p53 amyloid fibril structure and discuss the characteristics and effects of p53 amyloid aggregation, as well as the pathogenic mechanism leading to the occurrence of aggregation in tumors. Finally, we summarize the various inhibitors targeting p53 aggregation and prion-like behavior. In conclusion, a comprehensive understanding of p53 aggregation can expand our understanding of the causes leading its loss of physiological function and that targeting p53 aggregation might be a promising therapeutic strategy for tumor therapy.

## Background

Prion and prion like diseases, which result from the misfolding and aggregation of prion proteins, have been demonstrated in transmissible spongiform encephalopathies and various neurodegenerative diseases. The deposited key proteins can affect the function of the central nervous system or peripheral organs. Pathological aggregation of these key proteins can spread directly within or between cells, even between the same or different species of animals, leading to a gain of toxic function and ultimately to cell death [1–5]. In recent years, this concept has been extended to the field of oncology, and a variety of important proteins, such as the tumor suppressors p53 and PTEN, have been found to be pathologically aggregated in tumors, leading to functional alterations and tumor progression [6–8]. p53, as a well-known transcription factor regulating intracellular gene expression, has been extensively studied for its biological function for more than three decades. It has been found that malignant tumors characterized by p53 mutations may share a common propagation mechanism with neurodegenerative diseases [9–12], and p53 can aggregate into structures such as oligomers or amyloid fibrils, which in turn can affect its normal suppressor function [13–15].

To date, p53 aggregation has been found in various tumors, and wild-type p53 was found to be aggregated in neuroblastoma [16, 17], breast cancer [18], colon cancer [19], and retinoblastoma [20]. The accumulation of p53 aggregates was detected in paraffin-embedded breast tumor biopsies and basal cell carcinoma (BCC) samples by amyloid oligomer-specific antibody A11 or fibrillin-specific antibody OC. The colocalization of mutant p53 aggregates with amyloid oligomers in biopsied breast tissues was confirmed by immunofluorescence assays [18]. p53 may lose its suppressor function and acquire oncogenic function after the occurrence of amyloid aggregates [21–23]. Therefore, elucidating the oncogenic mechanism of p53 aggregation will be of great significance for tumor research. This paper reviews the form and role of p53 aggregation in tumors and discusses the mechanism of p53 amyloid aggregation through p53 mutation, domains, and isoforms. Inhibitors targeting p53 amyloid aggregation are also summarized. Altogether, it will be helpful in obtaining a comprehensive understanding of p53 aggregation in tumors.

## Characteristics and roles of p53 aggregation in cancer

### Characteristics of p53 aggregation

p53 oligomerization occurs mainly through its oligomerization domain (OD) under physiological conditions. p53 dimerization is a normal event, and tetramer is considered the major active transcriptional unit. In nontetrameric forms of p53 (monomers and possibly dimers), p53 exists in the cytoplasm. The tetramerized p53 localize in nuclear for transcriptional activation [24]. When DNA is damaged, p53 is tetramerized and accumulates rapidly [25]. p53 exists in a mixed oligomeric state, and its state varies greatly from cell to cell [22].The conformations of p53 are found in a variety of states ranging from active tetramers and octamers to amyloid fibrils and amorphous aggregates [26]. Similar to the heterogeneity of p53 intermediates (oligomers), the structure of mature amyloidogenic proteins is diverse [27]. The various states upon aggregation play different roles in diseases. It has been demonstrated that polymorphisms of aggregation may determine the variability of clinical features in neurodegenerative diseases; likewise, the diversity of p53 aggregation in tumors may change p53 function through various pathways [28]. Intermediates such as prefibrillar aggregates and soluble oligomers are the most potent mediators of cytotoxicity [15]. However, this is incompatible with the survival of tumor cells. In contrast, p53 amyloid aggregation is not toxic, and tumor cells use it to perform biological functions [22, 29]. Therefore, we are more concerned with amyloid aggregation of p53 in tumors.

**Fig. 1:**
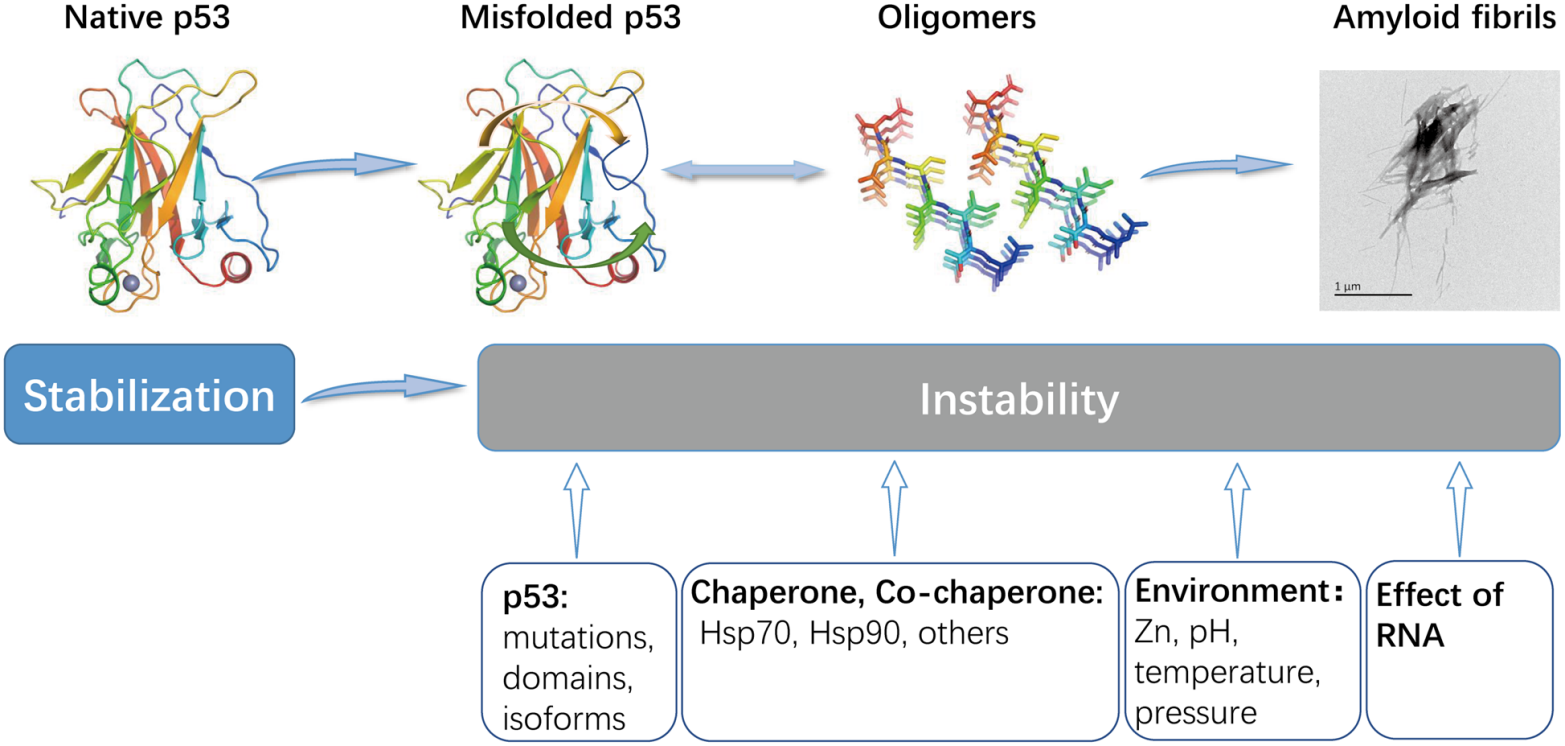
Illustration of p53 aggregation and the regulating factors. The scheme shows a potential route of aggregation, from the properly folded state, native state, to misfolded, aggregated forms of p53, including oligomers and amyloid fibrils. The main factors regulating p53 aggregation. (1) Some structural mutations, domains, and isoforms; (2) Chaperones, cochaperones, and some fiber stabilization factors are integrated into p53 amyloid aggregates, and abnormalities occur when they help p53-fold. (3) The protein state depends on the thermodynamic and kinetic factors in different environments. When the solution environment, such as Zn^2+^ concentration, pH, temperature, and pressure is changed, p53 may aggregate. (4) RNA molecules also modulate of p53 aggregation and seeding

Virtually, most proteins can aggregate under extreme chemical conditions, but only a small fraction of proteins aggregate in vivo under physiological conditions [30]. The amyloid aggregation of p53 has been extensively demonstrated by biophysical techniques such as X-ray diffraction and Fourier transform infrared spectroscopy (FTIR) [13]. As structural biology techniques advance, the first near-atomic resolution structures of amyloid fibrils have been achieved using cryo-electron microscopy and solid-state NMR spectroscopy in the last two years. Amyloid fibrils have a common architecture with β-strands in each protofilament aligned perpendicular to the long axis of the fiber, called cross-β amyloid folding, which is characterized by repetitive runs of approximately 4.7–4.8 Å along the protofibril axis [31]. This strong structure is considered a potentially primitive living structure due to its structural simplicity and ease of formation. Amyloid proteins can function in bacteria, fungi and even higher eukaryotes [32–34] but can also be associated with disease. Amyloid proteins share a common nucleation growth mechanism. First, monomeric protein precursors aggregate to form oligomers, which are dynamic, transient, heterogeneous, structurally unknown and variable [35–37]; oligomers can further produce higher-order substances that are essential precursors for amyloidogenic fibrils. During the process of self-assembly, each precursor undergoes a structural transition that leads to the formation of a β-strand-rich secondary structure, and once the cross-β structure of the fiber is formed, they can fragment, producing new protofibrils to recruit monomers. The protofibrils grow exponentially and eventually form a mature amyloid fibril structure [38] (Fig. 1).

### The roles of p53 amyloid aggregation

**Fig. 2:**
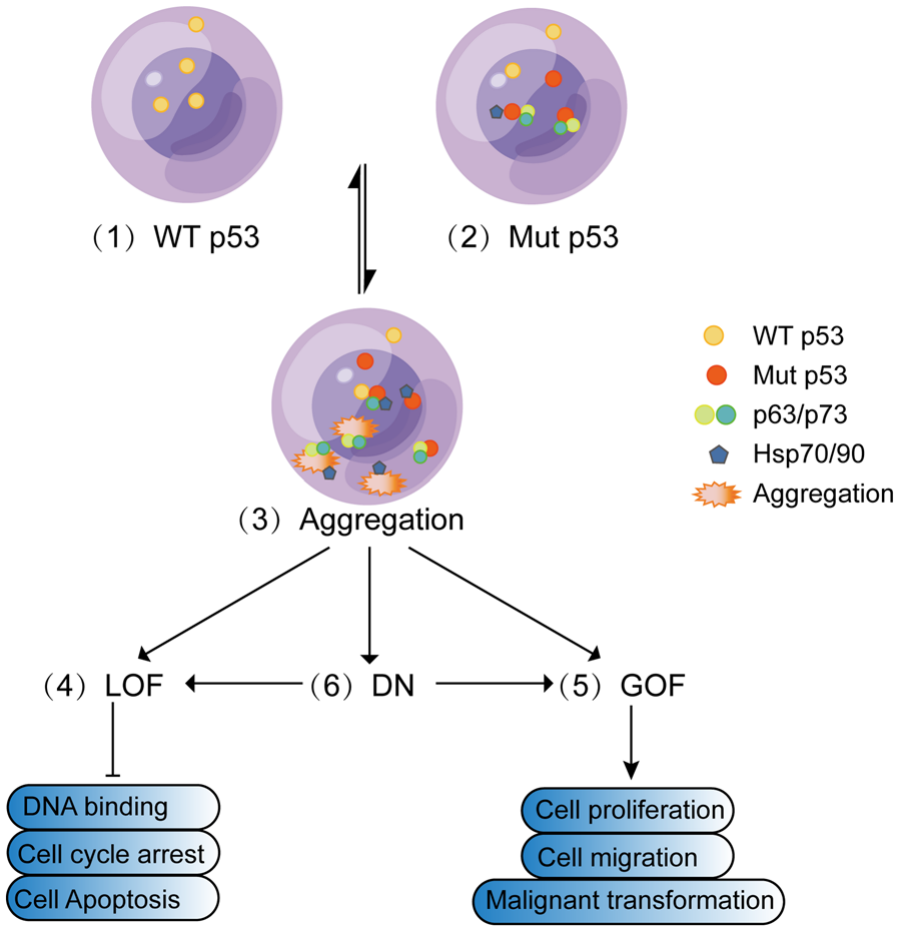
The roles of p53 aggregation in tumor progression. (1) In normal cells, wild-type p53 is functional at the nucleolus and can regulate the cell cycle and preserve cell integrity. (2) In cells expressing an aggregation-prone mutant p53, mutant p53 can interact with homologs p63/p73 or Hsp70/90. p53 will be inactivated due to genomic or cancer cell-specific mutation events. (3) Aggregated p53 may interact with different proteins, such as p63/p73 and heat shock proteins. p53 aggregation might lead to the following three kinds of effects. (4) Loss-of function [LoF]: Losing wild-type activity, p53 is no longer active in the nucleolus. (5) Gain-of-function [GoF]: Acquire oncogenic activity without disrupting the activity of wild-type p53. (6) Dominant-negative [DN]: Inhibit the wild-type p53 protein via a dominant-negative effect and display oncogenic activity (GoF) or no other activity (LoF)

p53 amyloid aggregation affects the normal function of p53 in several ways, and various independent studies have shown that the formation of mutant p53 aggregates is related to loss of function (LOF), gain of function (GOF), and dominant negative effect (DN) [11, 39, 40] (Fig. 2). It is important to elucidate the pathogenic mechanisms of p53 aggregation. If the sites and structures of p53 coaggregates are clear, it will be helpful to understand the function of p53 in tumor cells.

LOF: p53 amyloid aggregates are present in a variety of human and animal tumor tissues [15]. The loss of p53 suppressor function is obvious when p53 aggregates into large insoluble protein inclusions and prevents p53 from entering the nucleus. p53 amyloid aggregates not only prevent transport from cytoplasm to the nucleus but also escape proteasome degradation [8]. Even other proteins, including homologs of p53 and other tumor suppressors, are isolated by p53 aggregates and then deprived of their intrinsic cellular functions [22]. When p53 amyloid aggregates are formed in the nucleus, they are unable to bind DNA sequences for transcription, resulting in downregulation of antitumor genes and upregulation of the pro-cancer genes leads to loss of apoptotic and cell cycle arrest functions [8, 22].

GOF: alterations in p53 mutant function may be associated with the GOF of "emerging" target genes. For example, the first reported mutant, R175H, activated the promoter of multidrug resistance gene 1 (MDR1), which is not targeted by wild-type p53 [41]. Amyloid aggregation of mutant p53 is also associated with inflammation promotion and chemoresistance in glioblastoma [21, 29]. In 2013, Muller and Vousden proposed four types of mechanisms for stimulating GOF activity after p53 mutations and suggested the existence of a combination of two or more mechanisms leading to GOF [40]. For example, mutant p53 coaggregates with homologs p63/p73 or other transcription factors, the formed malignant signal increases the expression of p63-targeted genes, or the p53-p63 complex binds to rare DNA sequences to initiate the expression of other genes. Mutant p53 also coaggregates with heat shock protein 70 (Hsp70) [42] and the acetyltransferase p300 [43] and increases the expression of the antiapoptotic proteins Hsp70 and heat shock protein 90 (Hsp90) [22]. Studies in R248Q mutant mice revealed that R248Q mutant mice with a higher propensity to aggregate, compared to G245S or p53-deficient allele mice, could be more aggressive by seeding with wild-type p53 [44]. The latest paper indicated that p53 prion-like behavior leads to an alteration of the gene expression patterns, such as downregulate the key genes which involved in cycle checkpoints including *CDK6*, *CDK4*, *CDC45*, *PCNA*, *E2F2*, *RAD51*, *TIMELESS* and upregulate of genes including *ERBB2*, *MAP2K1*, *EGF.* As well as the EMT-associated genes, which contribute to tumor invasion and metastasis can be dysregulated by p53 amyloid formation [23]. Thus, p53 aggregation enable to promote cell proliferation, cell migration and malignant transformation. Tumors with a higher degree of aggregation are more likely to be aggressive [18].

DN effect: p53 acts as a tetramer in cells, and it is acknowledged that DN is caused by the combination of inactive mutant and wild-type p53 in a mixed tetramer, resulting in a reduced concentration of functional p53 in the cell. p53 aggregation provides an alternative hypothesis that the DN effect of conformationally unstable mutants is induced by mutation-induced coaggregation. When Milner et al. studied R248Q mutants, they found that the mixture of amyloid oligomers and protofibrils of R248Q, like seeds, accelerates the aggregation of wild-type p53, and the aggregation isolates not only functional p53 but also other tumor suppressors and p53 homologs [45]. The seeds of mutant p53 can lead to wild-type p53 or p63/p73 coaggregations and result in DN. It weakens the cellular defense against tumors and leads to impaired tumor suppressor function and enhanced tumorigenicity [22]. The aggregation propensity is different for p53 mutants, the conversion abilities of them as the seeds may be considered too, it may depend on the site and type of the mutation or others. Further study will be needed to confirm this hypothesis.

Actually, although LOF, GOF, and DN were tend to be studied separately, they are related to each other. In addition to losing the tumor-suppressing function of wild-type p53 (LOF), mutant p53 is also found to function in a tumor-promoting manner (GOF) through DN regulation of remaining wild-type p53 or independently of wild-type p53 [39, 40, 47]. In p53 monoallelic mutations, there is DN activity resulting in inactivation of the wild type, explaining its LoF [45]. What’s more, in recently published reaseach, Navalkar et al. using an in vitro model of full-length p53 amyloid, demonstrated the mechanism of LOF in conjunction with oncogenic growth of tumors (GOF) in p53. In addition to dysregulating genes associated with the cell cycle, proliferation, apoptosis and senescence formation, p53 amyloid formation alters the levels of p53 target proteins, which increases metabolism, enabling cells to survive [23].

### Mechanisms for aggregation

**Fig. 3:**
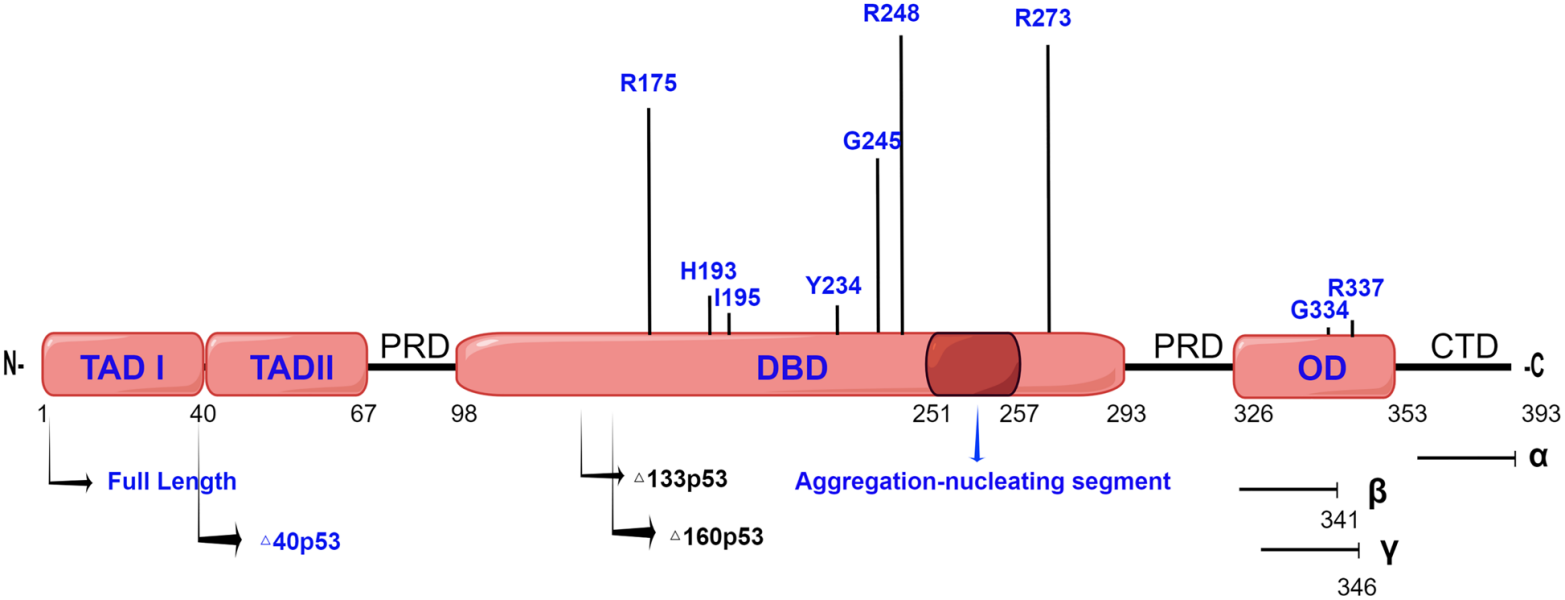
Different domains, mutants, and isoforms of p53 related to aggregation. Top: The sites of aggregation-related mutations are indicated by the corresponding residues. The bars above show the relative frequencies of missense mutations at the residues according to version R20 (July 2019) of the International Agency for Research on Cancer (IARC) tumor suppressor protein p53 (TP53) Database (http://www-p53.iarc.fr/). Middle: The frames show the different domains of p53 related to aggregation; the blue arrow shows the aggregation-nucleating segment. Bottom: The arrows indicate the start point (N-terminus) of the isoforms; the terminal arrows represent the C-terminal isoform variants. Transactivation domain I (TAD I); transactivation domain II (TAD II); proline rich domain (PRD); DNA-binding domain (DBD); oligomerization domain (OD); C-terminal domain (CTD)

Many mechanisms have been reported for p53 amyloid aggregation. In the following, we focus on the mechanism of p53 amyloid aggregation in terms of p53 high frequency mutations, structural domains, and isoforms (Fig. 3).

#### p53 mutants for amyloid aggregation

p53 mutations are common in tumors, and more than 200 different single-site mutations have been reported successively. R248, R175, G245, R273, R249, and R282 in the DNA binding domain (DBD) as the core region of p53 are taken for hotspot mutations [48]. Sites R248, R273 and R280 are called contact mutations [49]. These sites are critical for DNA binding, and mutations may impair the transcriptional activity of wild-type p53 but may not significantly affect the conformation of p53. Sites R175, G245, R248, R249 and R282 are known as structural mutations because of the importance for the structural stability of p53. A significant change in the structure of p53 may result in loss of affinity for its DNA binding elements [50]. p53 aggregation was detected in tumor samples or in vitro experiments with hotspot mutations. Furthermore, the distribution of p53 aggregation differed by mutation type. Overexpression of multiple mutant and wild type mutations in an osteosarcoma SaOS-2 cell line lacking endogenous p53 revealed that wild-type p53 and contact mutations were predominantly distributed in the nucleus, whereas structural mutations were mainly distributed in the cytoplasm [22]. The independent studies of p53 mutations are summarized in Table 1.

**Table 1:** Studies related to p53 aggregation

p53 domain	Aggregation related mutations or regions	Main content	Article Source
TAD (AA1–70)	Segment (AA1–63)	Under acidic conditions, it can aggregate into amyloid assemblies in vitro, and the aggregates are toxic to human SH-SY5Y cells	[51]
DBD (AA 94–293)	p53C (AA94–312)	Tendency to aggregate in vitro, same state of p53C and R248Q under pressure and high temperature; Exogenous addition of synthesized p53 core fibrils (seeds) in vitro can induce aggregation of the endogenous wild-type p53 in normal cells; In cellulo model of full-length p53 amyloid formation, the mechanism of loss of p53 tumorsuppressive function with concomitant oncogenic gain of functions, the mechanism of the transformation of cells due to p53 amyloids leading to cancer pathogenesis was established	[6, 23, 46]
	Segment (AA251–257)	The aggregation-nucleating segment is predicted to be a key region of p53 aggregation and has been confirmed by in vitro and in vivo experiments, and mutation I254R can inhibit aggregation	[8, 22, 52, 53]
	R175H	Weak oligomerization tendency in vivo; ApoDBD can initiate aggregation of zinc-bound DBD through the nucleation growth process	[18, 54]
	R248Q	Moderate oligomerization tendency in vivo; R248Q mutant aggregates exhibit GOF and DN effects	[13, 18]
	R273H	Strong tendency to aggregate in the body	[18]
	H193L, I195L, Y234C, G245S, wild-type	Small amounts of aggregates are present in the body, and tumor aggressiveness is strongly correlated with p53 aggregation	[18]
OD (AA324–355)	Full-length,N-terminal truncation (AA93–393)	After induction of aggregation in vitro, aggregates can penetrate cells through macrocytic drinking action and co-aggregation with cellular p53	[55]
	G334V	In vitro G334V peptide forms amyloid aggregates and hetero-oligomers with wild-type peptides through a two-step process	[56]
	R337H	In vitro R337H has a higher propensity to form amyloidogenic fibrils than wild-type p53	[57]

#### p53 domains for amyloid aggregation

Many studies have focused on the aggregation tendency of p53 domains. The three functional domains of p53, transactivation domain (TAD), DBD, and OD, have been demonstrated to form amyloid aggregates in vitro, and the highest aggregation propensity remains in the DBD region, which has the most hotspot mutations [13, 22, 46, 58]. In 2011, Jie Xu et al. found that the hydrophobic center of the DBD region has an aggregation-nucleating segment, which spans residues 251–257. It was experimentally confirmed that p53 mutations, leading to structural instability, increase the aggregation propensity by exposing this aggregation-nucleating segment, the exposed segment triggered coaggregation of wild-type p53 and its family members p63, p73 into cellular inclusions, caused various biochemical effects leading to gain of function. Finally, p53 achieves the functional transformation process from a suppressor gene to an oncogene. The hydrophobic residue isoleucine at site 254 is essential for p53 aggregation, as it is prone to aggregation [22]. Ghosh et al. also demonstrated the ease of segment aggregation by molecular dynamics (MDs) simulations. It was noted that mutations, intrinsic instability, or loss of Zn^2+^ of p53 leads to exposure of this segment, which can result in p53 self-aggregation. The process is an ordered process by which the aggregation-nucleating segment self-assembles into a nonnative state of β-structured molecular assemblies [53]. Furthermore, the amyloidogenic region of p53 (P8) was used as the amyloid seed to establish an "in-cell" model, which successfully led to the aggregation of natural p53 in cells to form amyloid fibrils, inactivating natural p53 and converting it into oncoprotein. The full-length and N-terminal truncated protein (p53C) aggregates can also be internalized into the cytoplasm and induced to coaggregates with endogenous p53 protein, which supports the hypothesis that p53 aggregates have prion properties [8].

However, by analyzing aggregates by restriction protein hydrolases and the effect of mutations on kinetics and agglomeration products, Wang et al. suggested that there is no unique aggregation sequence for p53 in vitro but rather multiple sites. The occurrence of p53 aggregation is not the contribution of an individual site but a collaborative network [59]. Stindt et al. also questioned the site I254 as a necessary site for coaggregation [60]. However, the denaturation status of p53 in vitro cannot comprehensively reflect the actual situation in cells and tissues. Therefore, whether there is a dominant core aggregation site or multiple site interaction, what each site performs, which mutations accelerate aggregation and their mechanisms need to be proven by further studies.

#### p53 isoform for amyloid aggregation

p53 dysfunction may also be caused by abnormal expression of isoforms. The p53 gene consists of 11 exons [61]. Through selective initiation of translation, selective promoter use and selective splicing, the p53 gene can theoretically be expressed in 12 different isoforms (p53α, p53β, p53γ, △40p53α, △40p53β, △40p53γ, △133p53α, △133p53β, △133p53γ, △160p53α, △160p53β, and △160p53γ) [58, 62, 63]. p53 isoforms are differentially expressed in tumors, and isoforms have diverse transcriptional activities and suppressor functions, which can affect various biological functions [64, 65]. Under normal conditions, as one of the cellular DNA damage stress responses, wild-type p53 tetramers are formed and bind to target sequences to activate target genes such as p21 [66] and BAX [67] during the tumor suppressor pathways [68–70]. In contrast, mutant p53 or others alter the expression of wild-type p53 or its isoforms, leading to aggregation and inhibiting its normal function [63].

As mentioned before, a partial peptide of the p53C structural domain can induce p53 aggregation in vitro, which is similar to the N-terminal truncated p53 isoforms (Δ40p53, Δ133p53, and Δ160p53), and isoforms can form a complex with full-length p53, leading to its inactivation [61, 63]. Although it has been hypothesized that some isoforms aggregate in tumor cells [71], less has been demonstrated experimentally. The presence of shorter p53 isoforms was found to be associated with chemoresistance in an early study of ovarian tumor resistance, and it was found that the presence of p53 aggregates affects the response of ovarian tumors to chemotherapy [72, 73], strongly suggesting a more robust relationship between p53 isoforms and aggregation. Another study of endometrial cancer (EC) first reported that cytoplasmic Δ40p53 is the major component of p53 amyloid aggregates in EC cells. The researchers examined the expression patterns of different p53 isoforms as well as their roles in the formation and localization of p53 amyloid aggregates in EC and nontumor cell lines. They discovered that full-length p53 and △40p53 were predominantly expressed in EC cells. Immunofluorescence revealed that △40p53 expression was mainly localized in the cytoplasm as punctate structures, which is similar to the solid morphology structures in neurodegenerative lesions. The △40p53 protein lacks the conserved N-terminal transcriptional domain TAD1 but still contains TAD2, and TAD1 significantly decreases the aggregation of the wild-type p53 DNA-binding domain, confirming that △40p53 has a higher aggregation tendency [74].

### Factors contributing to p53 amyloid aggregation

The kinetics of amyloid aggregation occur slowly with an extended lag period in vitro but accelerate dramatically once seeds are formed, and the thermodynamics are altered. In vivo, studies are much more complicated due to proteases, chaperone proteins, and some fibrillar stabilizing factors, such as extracellular matrix components and other proteins. They will incorporate into amyloid aggregates. Some factors that may contribute or accelerate the production of amyloid aggregates are as follows: the overproduction of wild-type amyloid [75], the breakdown of wild-type proteins into amyloid fragments [76], genetic or acquired mutations that alter the protein sequence [77]. p53 binds RNA specifically or nonspecifically, may also resulting in changes in their propensity to aggregate [78]. The main factors that regulate p53 aggregation are present in Fig. 2.

Mutations in key proteins: Conformational changes in key proteins lead to their pathological aggregation, ultimately resulting in deposits and disease, which is the unified pathogenesis of conformational diseases [79]. Mutations in key proteins are the most common cause of conformational diseases. For example, mutations in the Aβ precursor protein make it susceptible to cleavage and the formation of amyloid-based chains, increasing the risk of Alzheimer's disease [80, 81]. Another example is the A53T mutation of the α-synuclein gene, which occurs in a minority of patients with hereditary Parkinson’s disease [82]. The mutation disrupts the α-helix structure of α-synuclein and predisposes it to the β-sheet structure [83]. To some extent, the β-sheet structure is involved in the self-aggregation process of proteins and forms amyloid structures, resulting in plaques and tangles, which lead to the development of diseases [84]. Under normal circumstances, when cells are damaged or show a tendency to become cancerous, p53 activates the self-repair system in harmony, leading to cell apoptosis. In tumor cells, the cancer-associated p53 mutations reduce the stability of p53, unstable p53 cannot perform its normal function, and the cells will proliferate indefinitely, leading to tumorigenesis [85].

Chaperone abnormality: Protein folding is a prerequisite for normal function, while misfolding affects biological functions or even generates harmful oligomers. It is closely related to the binding of molecular chaperones and proteases in the intracellular translation process [86]. On the one hand, chaperones bind to the exposed hydrophobic surface of misfolded proteins to prevent aggregation and promote protein folding and assembly [87]; on the other hand, energy-dependent proteases remove irreversibly damaged proteins to maintain normal cellular function [88]. If the protection mechanism is impaired, for example, the exposed surface of the misfolded protein is not recognized by the molecular chaperone or protease, or the rate of polymerization is faster than chaperone/protease recognition, the misfolded protein that is neither protected by the molecular chaperone nor degraded by the protease may aggregate and lead to conformational disease [86].

Hsp, well-known chaperones, are frequently overexpressed in various tumors [89]. Protein denaturation and aggregation are potent triggers for their response. Hsp90 may help mutant p53 fold properly to survive without degradation in tumors, and accumulated p53 may acquire antiapoptotic properties by activating heat shock proteins [22]. Jie Xu et al. overexpressed wild-type and mutant p53 in the osteosarcoma SaOS-2 cell line and found that the overexpressed R273H (contact mutation) produced only similar chaperone levels to wild-type p53, but the overexpressed R175H (structure/aggregate mutation) induced a substantial upregulation of Hsp70 and Hsp90 [22].

Environment and other factors: polypeptide chains seem to have a universal ability to form amyloid fibrils, but different amino acid sequences have different propensities to form amyloid fibrils [90]. The state of polypeptide chains after ribosome synthesis depends on thermodynamic and kinetic factors in different environments [91]. Different solution environments, such as Zn^2+^ concentration, pH, temperature, and pressure, may destabilize the side chain interactions, the original folding structure may be opened, and a new structure or polymer may be formed under other conditions [92–95]. It is likely that the misfolded intermediates start with a mild conformational change, and the exposed hydrophobic groups make the intermediates difficult to dissolve in an aqueous environment. Then, the unstable intermediates interact with each other to be more stable. Furthermore, they lead to the formation of misfolded aggregates or amyloid fibrils [96]. p53 aggregation can be induced under low pH conditions, such as the TAD region being exposed to form aggregates in vitro [51], and the mutant R337H has a higher propensity to form amyloidogenic fibrils when the OD region is exposed to acidic pH or high temperature [57].

The effect of RNA: RNA molecules could modulate of p53 aggregation and seeding was reported recently [97]. It is possible that RNA-binding to the C-terminus of p53 can significantly affect its functional oligomerization and DNA interaction, which are two main preconditions for adequate transcriptional activity [98]. Furthermore, the protein: RNA molar ratio can directly affect the pathway of p53 aggregation [78]. The low ratio of RNA: protein is helpful for form large p53 aggregate, wheras high ratio prone to decrease the aggregation [97]. In addition, a liquid–solid phase transition has been proved to result in amyloidogenesis when protein and nucleic acids accumulate in condensates [99]. p53 can assemble membrane-less organelles formed by RNA molecules and disordered, low-complexity regions of RNA-binding proteins (RBPs) by the mechanism of liquid − liquid phase transitions (LLPT) [100].The concept that LLPT is composed of p53 and RNA, and the effect for cancer, need further research.

### Targeting p53 amyloid aggregation in cancer therapy

**Fig. 4:**
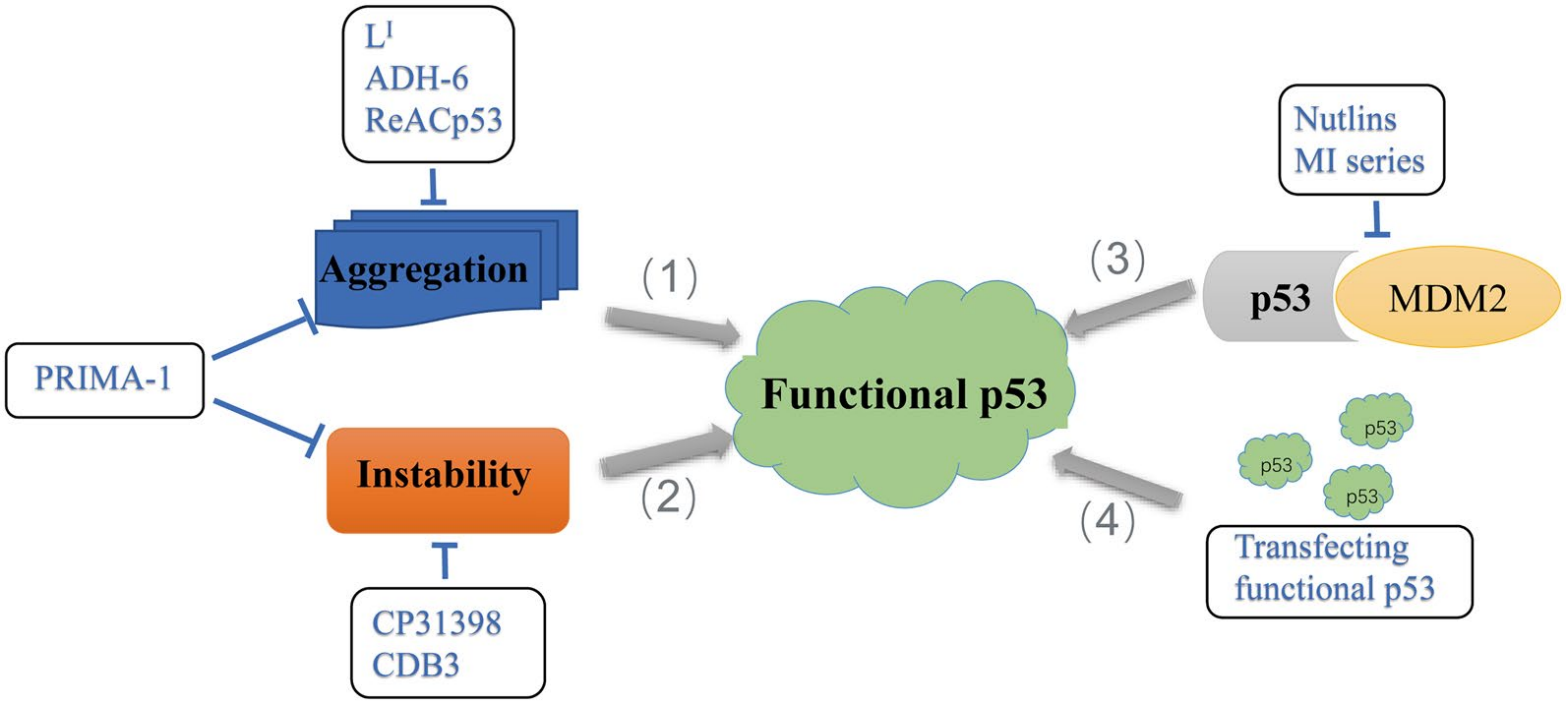
Strategies to recover p53 function in cancer therapy. (1) L^I^, ADH-6, ReACp53 can block p53 aggregation and prion-like behavior. (2) CDB3, CP31398 can stabilize p53 and restore the normal functionality. (3) Nutlins, MI series can interfere with the binding of p53-MDM2 and recover the function of p53. (4) Transfecting functional p53 via viruses to recover p53 function

As an important suppressor gene, p53 inactivation plays an important role in tumors, and scientists have investigated various approaches targeting p53 to recover the function of p53. For example, interfering with p53-MDM2 binding through small molecules or peptides [60, 89, 101], transfecting functional p53 via viruses [102], and restore the normal functionality of mutant p53, such as CDB3 [103], CP31398 [104, 105]. Among them, PRIMA-1 and its analog, used to restore the functionality of several conformation mutants [106, 107], were found to reverses mutant p53 aggregate accumulation [108](Fig. 4). The treatment of tumors as p53 prion-like diseases is still in the early stages, and it is valuable to design compounds to block p53 aggregation and prion-like behavior. The compounds can be natural or synthetic small molecules, peptides, protein mimetics, etc. They stabilize proteins and inhibit oligomerization and/or fibrillation. In addition, blocking the templating, amplification and other cell spreading of aggregated proteins are promising treatments. The existing inhibitors that can block p53 aggregation are summarized in Table 2.

**Table 2:** Inhibitors associated with blocking p53 aggregation

Inhibitor	Type	Targeting the p53 mutation site	Principle	Experiment
ReACp53[109]	A designed 17-residue peptide Inhibitor	Target R175 and R248 in HGSOC, have no effect on cells with folded wild-type p53	p53 amyloid spine structure is used to design ReACp53 ((R9)RPILTRITLE). Targeting p53 segment 251–257	In vitro and in vivo
L^I^ [110]	Bifunctional ligands	Y220C	Zn-free p53 exhibits accelerated protein aggregation, and L^I^ modulate mutant p53 aggregation and restore zinc binding using a metallochaperone approach	In vitro and in vivo
Tripyridylamide ADH-6[111]	Protein mimetic amyloid inhibitor	Target R175 and R248 in Pancreatic cancer, have no effect on cells with folded wild-type p53	α-Helix mimetics are small molecules that imitate the topography of the most commonly occurring protein secondary structure, serving as effective antagonists of protein–protein interactions (PPIs) at the interaction interface	In vitro and in vivo

Peptide inhibitor: The most notable therapy for targeting mutant p53 aggregation is ReACp53, which was developed by Wang et al. in 2016. ReACp53 is a sequence-specific peptide inhibitor that inhibits p53 mutant aggregation and tumor growth in a peptide-based approach. ReACp53 acts in the aggregation phase of p53 dynamic equilibrium to rescue the function of p53, and it targets the 252–258 region of p53. The hydrophobic isoleucine in this aggregation nucleating segment is replaced by an arginine in ReACp53, which inhibits the tendency of aggregation in this region by shielding the segment and converting the folding to a functional, wild-type-like state [109]. The inhibitor inhibited mutant p53 aggregation and tumor suppression and rescued the function of mutant p53 in human ovarian and prostate cancer cells, resulting in slower proliferation in vitro and tumor shrinkage in vivo. However, there are also limits, and the authors noted that if wild-type p53 partially unfolds and aggregates, the designer peptide may also bind to it. Thus, if this occurs in normal cells, there could be systemic toxic effects [109]. Guo et al. doubled the function of the peptide and proposed that the peptide may work through multiple pathways simultaneously. He argued that p53 did not contain a crucial target for certain anti-polymerization peptides in in vivo and in vitro experiments and questioned the possibility of their interaction [59].

Bifunctional small molecule: The DBD region of p53 contains a Zn^2+^ ion that is crucial for proper protein folding and function. Zinc-free p53 exhibits an accelerated aggregation of proteins [54, 112, 113]. Mutations in p53, such as Y220C, often result in the loss or alteration of Zn binding in the core, and the exposure and expansion of aggregation nucleating segments may lead to aggregation. L^I^ is a novel designed bifunctional ligand. On the one hand, iodine in L^I^ contributes to the interaction with the hydrophobic segment; on the other hand, L^I^, which possesses metal chaperone activity, can restore the binding of zinc in mutant p53. It was found that L^I^ not only restores the function of p53 by regulating the aggregation of mutant p53 and interacting with the zinc-binding fragment but also restores zinc binding using the metal chaperone approach, which greatly increases the content of zinc in cells. It increases cytotoxicity in tumor cells and ultimately restores p53 function. Combined with oxaliplatin, L^I^ increases the effectiveness of platinum-based therapy [110].

Protein mimics: Oligopeptide-based α-helical mimics have been reported to be effective in modulating the self-assembly of the key proteins, amyloid β-peptide in Alzheimer's disease [114, 115] and islet amyloid polypeptide in type II diabetes [116, 117] for a long time. α-Helical mimics are small molecules that mimic the topology of the most common protein secondary structures and act as effective antagonists of protein–protein interactions at the interface. The study confirmed that the small molecule amyloid inhibitor can be extended to the therapy of mutant p53 self-assembly. A tripyridylamide named ADH-6 was screened from an oligopyramide library to target and isolate the aggregated mutant p53 (R248W and R175H) in tumor cells and then restore the transcriptional activity of p53, resulting in cell cycle arrest and apoptosis. Moreover, ADH-6 was not toxic to healthy tissues, thus greatly prolonging survival. This study effectively established a bridge between amyloidosis and tumors [111].

Since p53 aggregation proceeds through multiple sites, blocking one site may not be an effective strategy to stop its aggregation. Combining physical, chemical, and computational approaches to obtain information about the initial steps of protein aggregation revealed the existence of a unique precursor state molten globular phase of p53 [118]. The protection of backbone hydrogen bonds (BHBs) by nonpolar amino acid side chain carbon atoms has been shown to be a key factor in keeping the protein core dry and maintaining protein stability [119]. Molecular dynamics (MD) simulations have been applied to identify potential defect sites, and the dynamics and hydration of p53 could be recovered through MD during drug development [120]. All these new techniques and perceptions provide clues and a theoretical basis for the design of more rational and effective drugs and inhibitors targeting p53 aggregation.

## Conclusions

In addition to pathogenicity, aggregation has been reported to be functional in bacteria, fungi, and higher eukaryotes. The prevailing view of protein aggregation in the field of neurodegenerative diseases is abnormal and harmful to cells. In 2018, a study reported that aggregates of the TDP-43 protein, which occurred in most cells of neurodegenerative diseases and are considered harmful, are beneficial to healthy muscle. We may change our mind that amyloid aggregation may have beneficial effects rather than simply being associated with disease [121].

To date, there is still much controversy regarding whether tumors can be classified as prion diseases, and many questions need to be addressed with experimental evidences. In addition to p53, scientists have also searched for the presence of other related proteins that misfold or aggregate in tumors. PTEN, which has properties similar to p53, such as a high propensity of mutation, intrinsically disordered regions (IDRs), and oligomerization ability, has been reported to have aggregation behavior recently [7]. Computational analysis also revealed that PTEN is prone to aggregation, which was subsequently confirmed in vitro. During tumor cell culture, protein homeostasis is severely dysregulated under stressful conditions, and mutant PTEN is prone to amyloid aggregation, similar to wild-type PTEN [122, 123]. In a small survey, aggregation was found in more than 25% of uterine tumor tissues, and PTEN aggregation status was negatively correlated with survival [124]. The retinoblastoma tumor suppressor (RB) also has aggregation properties. Substable structural domains A and B of RB are necessary for folding and stabilization, however, the AB domain is in a critically stable state, and mild perturbation destabilizes it, leading to oligomerization and partial aggregation [20].

Over the years, scientists have comprehensively investigated p53 in many aspects, and new p53 aggregation and prion-like characteristics in tumors have been proposed in recent years. The aggregated mutant p53 in cells, the interaction with its targets, the signaling with other cells, and the effects on tumor progression represent an emerging field and new therapeutic possibilities. Elucidation of the cellular biology and structural features of p53 amyloid aggregates will be an important topic in tumor biology and will provide new perspectives and directions for targeting p53 for tumor treatments.

## Data Availability

Not applicable.

## Abbreviations

BCC: Basal cell carcinoma
BHBs: Backbone hydrogen bonds
DBD: DNA binding domain
DN: Dominant negative
EC: Endometrial cancer
FTIR: Fourier transform infrared spectroscopy
GOF: Gain of function
Hsp70: Heat shock protein 70
Hsp90: Heat shock protein 90
IDRs: Intrinsically disordered regions
LLPT: Liquid − liquid phase transitions
LOF: Loss of function
MDR1: Multidrug resistance gene 1
MDs: Molecular dynamics
OD: Oligomerization domain
RB: Retinoblastoma tumor suppressor
RBPs: RNA-binding proteins
TAD: Transactivation domain
